# Preliminary report of de novo adipogenesis using novel bioabsorbable implants and image evaluation using a porcine model

**DOI:** 10.1007/s10047-022-01313-8

**Published:** 2022-03-02

**Authors:** Shuichi Ogino, Atsushi Yamada, Yusuke Kambe, Takashi Nakano, Sunghee Lee, Michiharu Sakamoto, Yuki Kato, Saki Okumura, Junko Okano, Koji Yamauchi, Yoshihisa Suzuki, Tetsuji Yamaoka, Naoki Morimoto

**Affiliations:** 1 Department of Plastic and Reconstructive Surgery, Shiga University of Medical Science, Seta Tsukinowa-Cho, Otsu, Shiga 520-2192 Japan; 2 Department of Research and Development for Innovative Medical Devices and Systems, Shiga University of Medical Science, Seta Tsukinowa-Cho, Otsu, Shiga 520-2192 Japan; 3 Department of Biomedical Engineering, National Cerebral and Cardiovascular Center Research Institute, 6-1 Kishibe-shimmachi, Suita, Osaka 564-8565 Japan; 4 Department of Plastic and Reconstructive Surgery, Graduate School of Medicine, Kyoto University, 54 Shogoin, Kawahara-cho, Sakyou-ku, Kyoto, 606-8507 Japan; 5 Gunze QOL Research Center Laboratory, 1 Zeze, Aono-cho, Ayabe, Kyoto 623-0051 Japan

**Keywords:** Adipogenesis, Animals, MRI, Ultrasound, 3D surface imaging

## Abstract

Our bioabsorbable poly-l-lactic acid (PLLA) mesh implants containing collagen sponge are replaced with adipose tissue after implantation, and this is an innovative method for breast reconstruction. In this preliminary study, we investigated the formation of adipose tissue and evaluated the process via multimodal images in a porcine model using an implant aggregate to generate the larger adipose tissue. The implant aggregate consists of PLLA mesh implants containing collagen sponge and a poly-glycolic acid woven bag covering them. We inserted the implant aggregates under the porcine mammary glands. Magnetic resonance imaging (MRI), ultrasonography (USG), and 3-dimensional (3D) surface imaging and histological evaluations were performed to evaluate the formation of adipose tissue over time. The volume of the implant aggregate and the formed adipose tissue inside the implant aggregate could be evaluated over time via MRI. The space within the implant aggregate was not confirmed on USG due to the acoustic shadow of the PLLA threads. The change in volume was not confirmed precisely using 3D surface imaging. Histologically, the newly formed adipose tissue was confirmed on the skin side of the implant aggregate. This implant aggregate has the ability to regenerate adipose tissue, and MRI is an appropriate method for the evaluation of the volume of the implant aggregation and the formation of adipose tissue.

## Introduction

The number of breast cancer patients is increasing. Mastectomy is associated with several problems in women, such as loss of femininity, fertility, charm, and sexuality [1, 2]. These have led to an increasing number of patients desiring breast reconstruction. Currently, reconstruction of the breast is performed via autologous tissue transfer, an artificial implant, and autologous fat transfer. The key components of aesthetic outcomes of breast augmentation are breast symmetry, size, and shape [3], and many techniques are reported to minimize operative complications while maximizing aesthetic appearance [4–7]. However, after breast reconstruction or breast augmentation, the size and shape of the reconstructed breast gradually change due to detumescence or atrophy. Therefore, evaluation of the reconstructed breast over time is required.

Both magnetic resonance imaging (MRI) and 3-dimensional (3D) surface imaging are accurate and reliable methods to assess breast volume [4, 8, 9]. Precise estimates of breast volume and detection of soft tissue can be achieved via MRI. However, repeated MRIs are not practical or cost-effective. In contrast, 3D surface imaging is able to be performed in an outpatient setting, making it less of a burden on the patient [10–12]. In a previous in vivo study, we confirmed the formation of adipose tissue using ultrasound imaging in the internal space of polypropylene mesh containing a collagen sponge (CS) 1 year after implantation [13].

Tissue engineering aims at the regeneration of tissues or organs through the combination of scaffolds, growth factors, and cells [14–16]. Adipose tissue has been reported to regenerate with the combination of a bioabsorbable scaffold, growth factors, and/or cells, such as adipose‐tissue-derived stromal cells (ASCs) or mature adipocytes [17–23]. Mechanical force also affects adipogenesis, and an in vitro study reported that adipogenesis from ASCs is inhibited by mechanical compressive force [24] or a mechanical stretch [25]. It is reported that the maintenance of the space by materials having the ability to withstand in vivo compressive forces for months leads to the regeneration of adipose tissue [26–28]. Large volumes of adipose tissue were regenerated via autologous fat transfer using bioabsorbable, polycaprolactone-based scaffolds in a porcine model [29] and poly-4-hydroxybutyrate mesh scaffolds clinically [30]. However, these methods that require autologous fat grafting have donor site morbidity. In addition, the safety of ASCs contained in the autologous fat for the breast reconstruction of post-breast cancer patients has not been confirmed because of the possible induction of breast cancer recurrence or metastasis [31–35].

We developed an innovative method of breast reconstruction using a bioabsorbable implant that is substituted by newly formed adipose tissue after implantation without the use of growth factors or cells [36, 37]. This implant aggregate consists of a poly-l-lactic acid (PLLA) mesh implant containing CS. The maintenance of the internal space in vivo for approximately 1 year after implantation is essential for the formation of adipose tissue using this PLLA implant in a rodent model [37]. However, the amounts of formed adipose tissue in rodent and rabbit models were not sufficient to fill the volume required for breast augmentation following clinical mastectomy [36, 37].

In this study, we inserted a PLLA implant aggregate enveloped with a poly-glycolic acid (PGA) mesh bag into the abdomen under the mammary gland using a porcine model. In this preliminary study, we evaluated the adipogenesis within this implant aggregate and investigated the efficacy of MRI, ultrasonography, and 3D surface imaging as evaluation methods of the adipogenesis over time.

## Materials and methods

### Ethics statement

The animals in this study were maintained at the Research Center for Animal Life Science, Shiga University of Medical Science. The experimental protocol was approved by the Institutional Animal Care and Usage Committee at Shiga University of Medical Science (Permit Number: 2019-7-10 (H3)) and was in accordance with the ARRIVE guidelines. The number of animals used in this study was kept to a minimum and all efforts to reduce animal suffering were made in accordance with the protocols established by the Research Center for Animal Life Science of Shiga University of Medical Science.

### Preparation of the bioabsorbable implants

The PLLA mesh implant containing CS (PELNAC^®^, Gunze Ltd., Tokyo, Japan) was prepared in a prolate spheroidal shape as previously reported [37]. Each columnar mesh was 1 cm in diameter and 1 cm in height and was knitted using a 2–0 PLLA thread (Gunze Ltd.). One side of the mesh was closed using purse string sutures. After tightly packing the mesh with 40 mm × 20 mm × 3 mm CS with a porosity of 80–95%, the other end of the mesh was closed using purse string sutures. The polar diameter of the implant was approximately 18 mm, and the greatest equatorial diameter of the implant was approximately 7.5 mm. The implants had multiple 1.5-mm square openings.

**Fig. 1:**
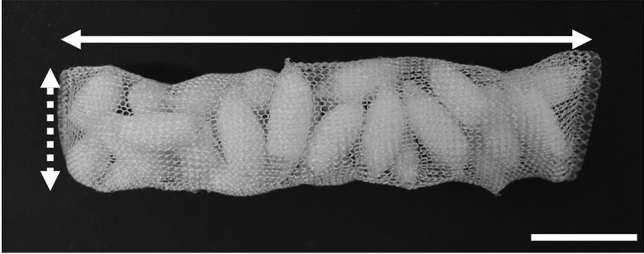
Poly-l-lactic acid implant aggregate. The gross appearance of the poly-l-lactic acid (PLLA) implant aggregate. The dashed white arrow indicates the largest diameter of the short axis, and the solid white arrow indicates the greatest length of the long axis. Scale bar: 2 cm

Next, 12–0.015-mm thick PGA multi-filaments were woven into an envelope-shaped bag. We prepared two flat PGA-woven bags that were 110 mm × 35 mm. Thirty pieces of the implant were packed into each PGA-woven bag, and two pieces of the implant aggregate were shaped into a cylinder with a diameter of 2.4 cm and a height of 9.0 cm (Fig. 1).

### Animal experiment

#### Experimental design and operative procedure

A minipig, CLAWN miniature swine, was chosen for this study due to its large body surface and skin that is similar to human skin. One 10-month-old female CLAWN miniature swine was purchased from the Kagoshima Miniature Swine Research Center. After a 2-week preservation period, the minipig weighed 26.3 kg. The animal was cared for as outlined in the Public Health Services Policy on Humane Care and Use of Laboratory Animals. General symptoms such as feeding status and limping were assessed daily.

**Fig. 2:**
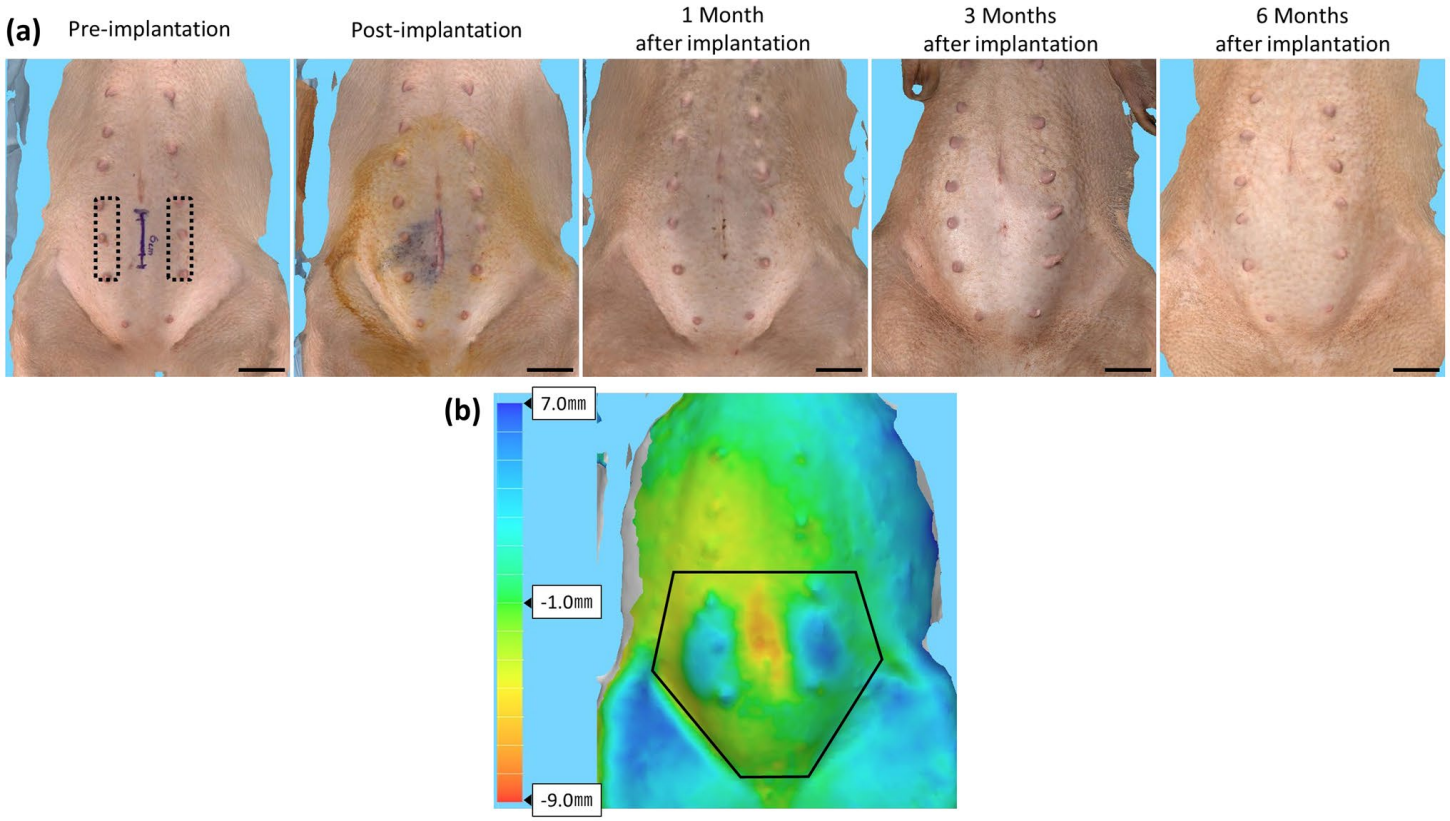
Three-dimensional imaging evaluation. **a** The 3D imaging at the abdominal side shows a slight bulge at 1, 3, and 6 months after implantation. The blue line represents the 6-cm incision. The black-dotted area shows where the implant aggregate was inserted. Scale bar: 5 cm. **b** The change in post-implantation compared to that in pre-implantation is shown. The black line indicates the area of the evaluated volume change. The volume in post-implantation increased 33.15 cm^3^ comparing with that in pre-implantation. The blue side means the protrusion, and the red side means the depression

The minipig was not fed overnight prior to the implantation. Sedation was achieved via an intramuscular injection of 25 mg/kg ketamine (KETALAR^®^, DAIICHI SANKYO Co., Ltd., Tokyo, Japan) and 0.02 mg/kg medetomidine (DOMITOR^®^, Nippon Zenyaku Kogyo Co., Ltd., Fukushima, Japan). After sedation, a peripheral intravenous line and a tracheal tube were inserted. The minipig’s SpO_2_ was monitored, and the minipigs were anesthetized by the inhalation of a mixture of air and oxygen containing 2.0–2.5% isoflurane. After shaving the abdominal region, a 6-cm midline incision was made in the abdomen starting 1 cm caudal to the umbilicus (Fig. 2a). Next, the fat tissue was incised, and a pocket was prepared over the fascia on each side of the abdomen. Once the implant aggregates were placed into the pockets, the fat and skin were closed with 2–0 blade nylon sutures (Nurolon: Johnson & Johnson K.K., Tokyo, Japan). After this study period, the minipig was used for an additional study.

#### Evaluation of the implant aggregates

The area of implantation and the appearance of the implant aggerates were evaluated under general anesthesia using MRI, ultrasound, and 3D surface imaging prior to implantation, immediately after implantation, and 1, 3, and 6 months after implantation. The tissue from just below the skin to the fascia, including the implant aggregate, on the right side of the abdomen was harvested 6 months after implantation. The implant aggregate in the left side of the abdomen was preserved for the next study.

##### Three-dimensional surface imaging protocol

Three-dimensional surface images were obtained using 3D imaging solution (Vectra H2: Integral Corporation., Tokyo, Japan). The volume change of the implantation area between pre- and post-implantation was assessed from the cranial edge of the umbilicus to the inguinal ligament (Fig. 2b).

##### Ultrasonography protocol

An ultrasonographic system (ACUSON S2000 HELX Evolution: Siemens Healthcare K.K., Tokyo, Japan) with a 9L4 probe were used to evaluate the tissue formation inside the implant aggregate.

##### MRI procedure

The minipig was placed on the examination table in the MRI room in the supine position. The mini pig’s abdominal part was scanned by a wide-bore 3 Tesla (T) MRI scanner (Magnetom Verio 3 T, Siemens Healthcare, Erlangen, Germany) using a dedicated 4ch large flex coil. The scan acquired weighted image and water excitation (WE) images in the transverse plane using T1-weighted Dixon imaging (TR/TE = 5.26/2.46 ms; flip angle = 10°; acquisition matrix = 352 × 172; field of view (FOV) = 285 × 350 mm^2^; slice thickness = 1.0 mm). During the scanning, breathing was controlled using an intravenous injection of 0.4 mg/kg rocuronium bromide (ESLAX^®^, MSD K.K., Tokyo, Japan). The obtained images were loaded to a 3D slicer [38], a software package for the analysis of medical images, to calculate the volume of the implant aggregate and the volume of the newly formed adipose tissue inside the implant aggregate.

#### Histological assessment of the newly formed tissues inside the implant aggregate

The specimen was harvested 1 cm away from the outer edge of the implant aggregate, including the fascia in contact with the implant aggregate. The harvested specimen was fixed with 4% paraformaldehyde phosphate buffer solution (FUJIFILM Wako Pure Chemical Corporation, Osaka, Japan). The specimen was divided into four equal blocks along the long axis of the implant aggregate. The second block from the caudal side was embedded in optimum cutting temperature compound (Sakura Fine Technical Co. Ltd., Tokyo, Japan) and frozen in ethanol dry ice prior to oil-red-o staining. The three remaining blocks were paraffin-embedded for subsequent hematoxylin–eosin (H&E), azocarmine and aniline blue (AZAN), and immunohistochemical staining. The 16-μm-thick frozen section from the central region of the tissues was prepared for oil-red-o staining. The 5-μm-thick sections from three aspects of the specimen were prepared for H&E, and the 5-μm-thick sections at the central aspects of the specimen were prepared for AZAN and immunohistochemical staining. Immunohistochemical staining of CD31 was performed to evaluate the formation of new capillaries in the newly formed tissue. After the deparaffinization and rehydration of the 5-mm-thick paraffin sections, they were immersed in diluted target retrieval solutions (415211; Nichirei Biosciences Inc., Tokyo, Japan) and incubated for 20 min at 98 °C. After being cooled to room temperature, the sections were rinsed once in distilled water and immersed in 3% hydrogen peroxide (FUJIFILM Wako Pure Chemical Industries Ltd.) and methanol (FUJIFILM Wako Pure Chemical Industries Ltd.) for 10 min to block endogenous peroxidase activities. The sections were then rinsed in distilled water and 50 mM Tris-HCI buffered saline (Takara Bio Inc., Kusatsu, Japan) with 0.05% Tween 20 (FUJIFILM Wako Pure Chemical Industries Ltd.) and 0.15 M NaCl (TBST). To block nonspecific protein binding, 3% bovine serum albumin (BSA) diluted with phosphate-buffered saline (PBS) was applied for 60 min at room temperature. A monoclonal mouse anti-rabbit CD31 antibody (ab182981: Abcam plc., Tokyo, Japan) was applied as the primary antibody at a dilution of 1:10,000 using 1% BSA in PBS and incubated overnight at 4 °C. The sections were rinsed in TBST. Next, a peroxidase-labeled secondary antibody (rabbit anti-goat simple stain MAX PO [R]; Histofine; Nichirei Biosciences Inc.) was applied for 30 min at room temperature. Sections were then rinsed with TBST and exposed to 3,30-diaminobenzidine tetrahydrochloride (Dako Japan Co., Ltd., Tokyo, Japan) for 5 min at room temperature and counterstained with hematoxylin. All microphotographs were obtained using a light microscope (IX83: Olympus corporation, Tokyo, Japan) at 40 × magnification. The newly formed tissues and the newly formed adipose tissue inside each implant were evaluated on the microphotographs of the H&E-stained sections using ImageJ software version 1.53 g (National Institutes of Health, Bethesda, Maryland, USA). The data were expressed as mean and range.

## Results

### 3D surface imaging findings

The 3D surface images are shown in Fig. 2a. At 1, 3, and 6 months after implantation, a slight bulge was observed at the abdominal surface. The pre- and post-implantation images are compared in Fig. 2b. The volume at the abdominal surface increased by 33.15 cm^3^ immediately after implantation.

### Ultrasonographic findings

**Fig. 3:**
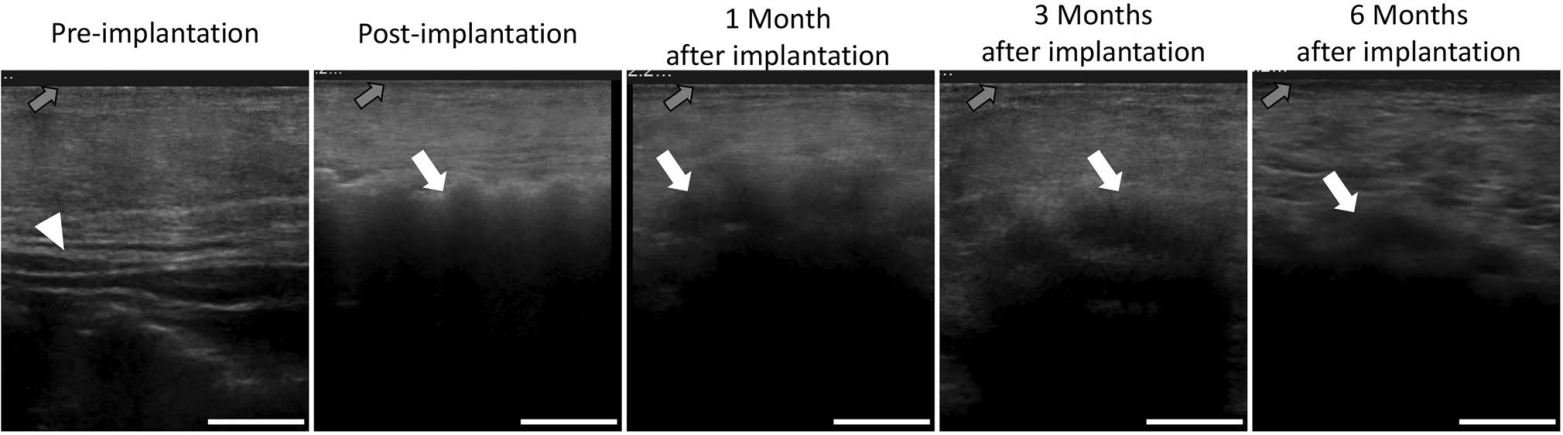
Ultrasound evaluation of the implant aggregate. The outer surface of the implant aggregate was defined immediately after implantation, but became less distinguished over time. The acoustic shadow of the outer surface diminished over time. The hyperechoic area was confirmed in the internal space of the implant aggregate 1 month after implantation. The white arrowhead indicates the deep fascia. The white arrows indicate the outer surface of the implant aggregate. The gray arrows indicate the epidermis. Scale bar: 1 cm

The ultrasonographic findings are shown in Fig. 3. The outer surface of the implant aggregate was hyperechoic immediately after implantation and was clearly defined. However, at 1, 3, and 6 months after implantation, the outer surface of the implant aggregate was not clearly defined. The ultrasonic observation of the internal space of the implant aggregate was disturbed by the acoustic shadow of the PLLA thread and PGA mesh immediately after implantation. At 1, 3, and 6 months after implantation, the acoustic shadow was diminished, and a hyperechoic area that represented the newly formed tissue or the PLLA thread was identified at the skin side of the implant aggregate.

### MRI findings

**Fig. 4:**
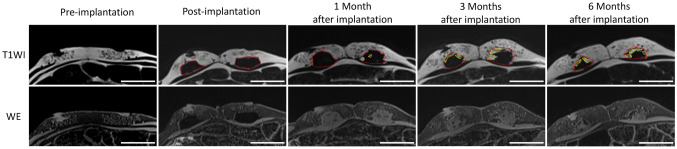
Magnetic resonance imaging evaluation of the volume of the implant aggregate and the newly formed adipose tissue. The red dotted lines indicate the area of the implant aggregate, and the yellow dotted lines indicate the area of newly-formed adipose tissue. The areas of the implant aggregates were confirmed at all time points. The newly formed adipose tissue was identified as hyperintense in the T1-weighted images and the hypointense in water excitation images at 1, 3, and 6 months after implantation. Scale bar: 5 cm

The MRIs are shown in Fig. 4. In the T1-weighted image (T1WI), the normal adipose tissue and the implant aggregate were able to be distinguished at all time points. The newly formed adipose tissue was identified as a high-intensity lesion in T1WI images and a low-intensity lesion in WE images. It was confirmed to be inside the implant aggregate especially at the skin side and in contact with the original porcine fat at 3 and 6 months after implantation. The time course of the volume of the implant aggregate and the formed adipose tissue inside the implant aggregate are shown in Table 1. The volume of the implant aggregate gradually decreased over time while the volume of adipose tissue inside the implant aggregate gradually increased over time. The newly formed adipose tissue occupied approximately 22% of the implant aggregate volume.

**Table 1:** Volume of the implant aggregate and the newly-formed adipose tissue

	Time after implantation			
	Post-implantation (*N* = 2)	1 Month (*N* = 2)	3 Months (*N* = 2)	6 Months (*N* = 2)
Implant aggregate (cm^3^)	28.35 (28.23–28.49)	24.49 (24.39–24.58)	20.86 (18.96–22.77)	18.00 (16.48–19.50)
Newly-formed adipose tissue (cm^3^)		0.01 (0–0.02)	0.43 (0.32–0.54)	3.94 (3.52–4.36)

Data expressed as mean (range)

### Histological assessment of the newly formed tissues

**Fig. 5:**
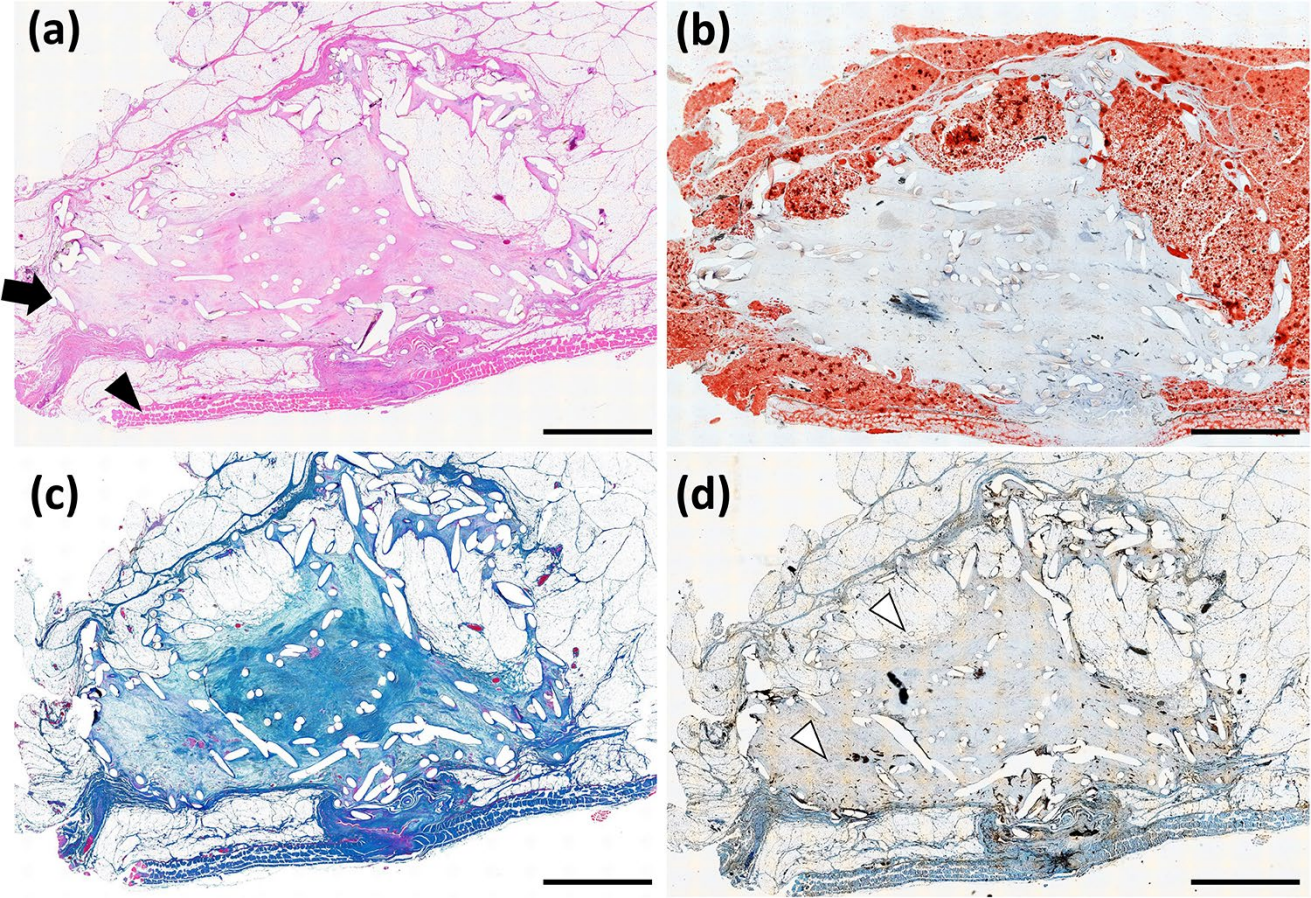
Histological evaluation of the implant aggregate. Light micrographs of **a** hematoxylin–eosin (H&E)-stained sections, **b** oil-red-o-stained sections, and **c** azocarmine and aniline blue (AZAN)-stained sections 6 months after implantation are shown. The presence of poly-l-lactic acid (PLLA) mesh is confirmed. The internal space in the implant aggregate is maintained, and the regeneration of adipose tissue and collagen fiber is confirmed. **d** Light micrographs of anti-CD31 are shown. Newly formed capillaries are observed both in the area of the newly formed adipose tissue and in the area of formed collagen fiber. The arrowhead indicates the fascia, the solid arrow indicates the PLLA thread, and the open arrow indicates the newly formed capillaries. Scale bar: 5 mm

No inflammation was observed around the harvested tissue. Micrographs of H&E-stained sections, oil-red-o-stained sections, and AZAN-stained sections are shown in Fig. 5a–c, respectively. The presence of PLLA mesh was confirmed histologically at 6 months after implantation. The internal space in the implant aggregate was maintained until 6 months after implantation, and the newly formed tissue inside the implant aggregate consisted of adipose tissue and collagen fiber. Most of the adipose tissue was on the skin side. However, if the implant was in contact with the adipose tissue, a slight amount of adipose tissue formation was confirmed on the fascia side as well. A micrograph of anti-CD31 stained sections is shown in Fig. 5d. The formation of capillaries was confirmed both in the area of the newly formed adipose tissue and in the area of formed collagen fiber.

The average internal area of the implant aggregate was 206.2 mm^2^, and that range was 126.9–267.1 mm^2^. The average area of newly formed adipose tissue was 65.4 mm^2^, and that range was 31.8–83.6 mm^2^.

## Discussion

The formation of adipose tissue inside the implant aggregate was evaluated and methods of the evaluation of adipogenesis were investigated using a porcine model. Newly formed adipose tissue was confirmed to be inside the implant aggregate at 6 months after implantation. MRI was determined to be an appropriate tool for the evaluation of the volume of the implant aggregate and the formation of adipose tissue.

The time course of the survival rate of transferred adipose tissue after breast reconstruction and breast augmentation can be evaluated using 3D surface imaging [39]. During 3D surface imaging of the breast, it is essential that the body size does not change during the observation period and that the sternum and costal cartilage are used as deep organ markers. In this porcine model, the implantation was performed in the abdomen due to the lack of sufficient space in the breast and the lack of thick adipose tissue mimicking the mammary gland in the back. However, in the abdomen, this implant aggregate protrudes to the outside of the abdominal cavity. In addition, as the body size increases, the adipose tissue becomes thicker. Therefore, the data regarding the original volume of the implant aggregate and the change in volume over time was not available in this study.

Ultrasonography is an easy-to-use imaging method. In this study, all tissues inside the implant aggregate were not confirmed within 6 months after the implantation due to the acoustic shadow of the PLLA thread and the PGA mesh on ultrasound. In our previous report, the space or the formed adipose tissue inside the polypropylene mesh was confirmed using ultrasound imaging after implantation [13]. These contrasting results may be due to the difference of the material or thickness of thread in the two studies. In PBS at 37 °C, PLLA was degraded to 50% by weight within 2 years and PGA was completely degraded within 2–3 months [40]. This implant needs to be filled with the formed adipose tissue inside at least 1 year [36, 37]. Therefore, in the next experiment, we need to investigate the regeneration of adipose tissue over a year after implantation using ultrasonography.

MRI is a non-invasive imaging method that does not expose patients to radiation and provides superior images compared to computed tomography. MRI is useful for the detection of soft tissue. In this porcine model study, we found that MRI was an effective method for the evaluation of the volume of the implant aggregate over time and for the confirmation of the newly formed adipose tissue inside the implant aggregate.

In our previous in vivo study, we reported the importance of maintaining the internal space for adipogenesis [36, 37]. Other studies have reported that the maintenance of the internal space using bioabsorbable materials leads to the regeneration of large volumes of adipose tissue with fat injection [29, 30]. Our implant aggregate has the ability to regenerate adipose tissue without the addition of growth factors or cells. Therefore, this is a novel bioabsorbable implant aggregate. Regarding the mechanism of adipose tissue formation inside the implant aggregate, ASC is supplied from the original adipose tissue around the implant aggregate, and mesenchymal stem cells are supplied from the newly formed capillaries. The histological (Fig. 5b) and MRI (Fig. 4) findings show that our implant aggregate regenerated the adipose tissue, especially in contact with the original adipose tissue on the skin side. Therefore, it is supposed that the ASCs supplied from the original adipose tissue are a key factor for the regeneration of the tissue.

The ultimate goal of this study was to investigate the correlation between the volume of the implant aggregate and the newly formed adipose tissue. The CS in vivo converts into regenerated connective tissue in 2 or 3 weeks. The degradation rate of PGA is 100% within 2–3 months under investigation for medical application [40]. The decrease in the ability to maintain the internal space due to the absorbance of the PGA and PLLA components might have led to the decrease of the volume of the implant aggregate over time. Whereas, the newly formed adipose tissue was histologically 36% of the volume of the implant aggregation. Angiogenesis is an important factor for the tissue regeneration. In this study, at 6 months after operation, the adipose tissue was regenerated from the skin side, and newly formed capillaries were found in the collagen fiber within the implant aggregate. A future study with a larger sample size and a longer follow-up is required.

## Conclusions

Our novel implant aggregate regenerates the adipose tissue. The volume of the implant aggregate and the adipose tissue can be evaluated using MRI.
