# Correction: Preliminary report of de novo adipogenesis using novel bioabsorbable implants and image evaluation using a porcine model

**DOI:** 10.1007/s10047-022-01374-9

**Published:** 2022-12-07

**Authors:** Shuichi Ogino, Atsushi Yamada, Yusuke Kambe, Takashi Nakano, Sunghee Lee, Michiharu Sakamoto, Yuki Kato, Saki Okumura, Junko Okano, Koji Yamauchi, Yoshihisa Suzuki, Tetsuji Yamaoka, Naoki Morimoto

**Affiliations:** 1 Department of Plastic and Reconstructive Surgery, Shiga University of Medical Science, Seta Tsukinowa-Cho, Otsu, Shiga 520-2192 Japan; 2 Department of Research and Development for Innovative Medical Devices and Systems, Shiga University of Medical Science, Seta Tsukinowa-Cho, Otsu, Shiga 520-2192 Japan; 3 Department of Biomedical Engineering, National Cerebral and Cardiovascular Center Research Institute, 6-1 Kishibe-shimmachi, Suita, Osaka 564-8565 Japan; 4 Department of Plastic and Reconstructive Surgery, Graduate School of Medicine, Kyoto University, 54 Shogoin, Kawahara-cho, Sakyou-ku, Kyoto, 606-8507 Japan; 5 Gunze QOL Research Center Laboratory, 1 Zeze, Aono-cho, Ayabe, Kyoto 623-0051 Japan

## Correction: Journal of Artificial Organs (2022) 25:245–253 10.1007/s10047-022-01313-8

In the article titled “Preliminary report of de novo adipogenesis using novel bioabsorbable implants and image evaluation using a porcine model,” (Ogino et al., 2022) the authors found erroneous descriptions of magnetic resonance images. They should be described as in this erratum.

1. In the Materials and methods section, the third sentence of the MRI procedure subsection should have been written as follows.
  The images were scanned in the transverse plane using 3D T1-weighted gradient-echo 2-point Dixon imaging (TR/TE = 5.26/2.46 ms; flip angle = 10°; acquisition matrix = 352 × 172; field of view (FOV) = 285 × 350 mm^2^; slice thickness = 1.0 mm). In addition, to acquire each TE image, this Dixon imaging method additionally calculated fat-only and water-only images.
2. In the Results section, the second and third sentences of the MRI findings subsection should have been as follows.
  In the fat-only images, the normal adipose tissue and the implant aggregate were able to be distinguished at all time points. The newly formed adipose tissue was identified as a high-intensity lesion in the fat-only images and a low-intensity lesion in the water-only images.
3. Figure 4 should have been as follows.

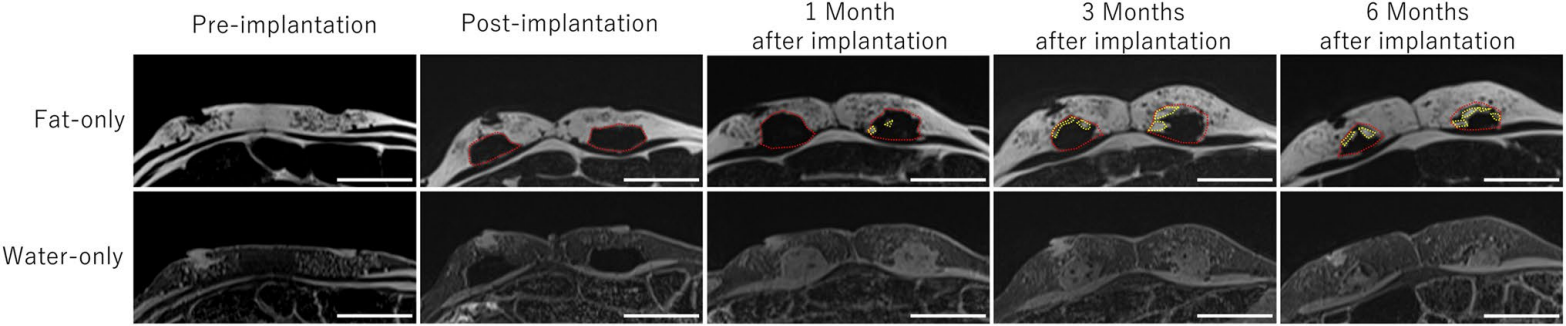

4. The fourth sentence of the figure legend for Figure 4 should have read as follows.
  The newly formed adipose tissue was identified as hyperintense in the Dixon fat-only images and as hypointense in the Dixon water-only images at 1, 3, and 6 months after implantation.

The authors apologize for these mistakes and any inconvenience they may have caused.

